# Factors associated with rural-urban safe disposal of children stools in Ghana

**DOI:** 10.1186/s13104-024-06701-2

**Published:** 2024-02-20

**Authors:** Martin Wiredu Agyekum, Florence Esi Nyieku, Sylvia Boamah Yeboah, Faustina Frempong-Ainguah

**Affiliations:** 1 Institute for Educational Research and Innovation Studies (IERIS), University of Education, Winneba, Ghana; 2 Department of Environmental Health and Sanitation Education, University of Education, Winneba, Ghana; 3 Faculty of Human and Social Studies, Mykolas Romeris University, Vilnius, Lithuania; 4 Regional Institute for Population Studies, University of Ghana, Legon, Ghana; 5 Ghana Statistical Service, Accra, Ghana

**Keywords:** Stool, Safe disposal, Rural-urban differentials, Children under 2 years, Ghana

## Abstract

**Introduction:**

The burden of children’s disease in many low-and middle-income countries is associated with poor sanitation, including unsafe disposal of children’s stool. Infants and toddler stools pose a greater public health risk than adults. Studies on stool disposal in Sub-Saharan Africa (SSA) and Ghana have focused on prevalence, patterns, and associated factors. Nevertheless, these studies have not focused on factors that independently influence rural and/or urban child stool disposal. This study, therefore, examines factors associated with safe child stool disposal in rural areas separately from urban areas towards Ghana’s readiness for ending open defaecation by 2030.

**Methodology:**

We examined young children’s faecal disposal drawing on the sixth round of the nationally-representative Ghana Multiple Indicator Cluster Survey (MICS) conducted in 2017/18. This study was restricted to children under two years, yielding a sub-sample of 3,476. Responses of caregivers or mothers who disposed of children less than two years faecal matter, their characteristics in addition to the child’s age in months were analysed. A binary logistic regression was used to examine the factors associated with the safe disposal of young children’s stools.

**Results:**

In the aggregated data, only 22% of households, regardless of their residence, dispose of their young children less than two years stools safely. From the disaggregated data, the rural analysis shows that 26% of young children’s stools were safely disposed of, compared to 16% in the urban analysis. The urban analysis shows that the child’s age, sex and caregiver’s marital status were significantly associated with safe disposal of stools. On the other hand, child’s age, caregiver listening to radio and household access to improved toilet facilities were significant in the rural analysis.

**Conclusion:**

The safe practice of stool disposal was very low. The results of this study show that urgent and different policies and strategies are needed to address child stool disposal in urban residences compared to rural residences if we are to meet SDG targets of ending open defaecation.

## Introduction

Safe disposal of young children’s stool prevents oral-faecal diseases [1, 2]. Young children, usually less than five years in low-income countries, defaecate in their close environment [1, 2]. This is because of the type of drop-hole and/or toilet facility available to the households [3]. Globally, about 616 million people in 2020 were using unimproved facilities, while 494 million practice open defecation, with nearly half of them living in sub-Saharan Africa (SSA) [4]. Open defecation is more prevalent in rural than urban areas [4, 5]. Unsafe disposal of children’s stools poses a greater public health risk due to the presence of higher pathogens which may be associated with cholera, diarrhoea and dysentery and even result in death [1]. The burden of children’s disease in many low-and middle-income countries is associated with poor sanitation, including unsafe disposal of children’s stool [3]. The disparity in access to safe and hygienic waste disposal methods has significant implications for child health [6, 7]. The Sustainable Development Goal (SDG) 6.2 aims to “achieve access to adequate and equitable sanitation and hygiene for all and end open defecation by the year 2030”, while SDG 3.9.2 aims to reduce “mortality rate attributed to unsafe sanitation and lack of hygiene services” [8]. However, progress towards these has been uneven across and within countries [9]. In Ghana, a number of strategies and programmes such as the Water, Sanitation and Hygiene Sector Development Programme (GWASHSDP) 2021–2030 seeks to improve sanitation and hygiene services to all Ghanaians irrespective of one’s physical location and socio-economic status. The key component is the safe and hygienic management and disposal of human excreta (including children’s stool). Achieving these goals requires the various dimensions of sanitation, including safe stool disposal and elimination of open defecation, are given the needed attention [10–13].

Excreta disposal is a service, not just an infrastructure; for children two years and below, this service is usually provided by the mother or caregiver [14–17]. Unsafe or improper disposal of young children’s faeces present a significant source of exposure to health risks. The ‘where’ and the ‘how’ these faeces are disposed off depend on several factors, including the age of the child as well as demographic and socio-economic aspects of the caregiver [18, 19]. This study adopts the socio-ecological framework, recognising that the disposal of a young child’s stool is a complex behaviour influenced by many factors. These encompass personal elements, interpersonal dynamics, household structures, community norms, and policy considerations [20, 21].

Many sanitation interventions in lower-middle-income countries, including Ghana, have focused more on the household solid waste disposal and ending adult open defecation practices in general with not much emphasis on young children’s stool disposal [21–23]. This neglect will in the long run not inculcate good practices among these children. However, non-hygienic disposal of children’s stool could be a major source of faecal contamination in the household environment [24–29]. For instance, children crawl, play and pick items from the ground into their mouths, exposing them to diseases.

Studies on stool disposal in SSA, including Ghana, have focused on prevalence, patterns, and associated factors [17, 30–34]. Nevertheless, these studies have not focused on factors that independently influence rural and/or urban child stool disposal. The 2021 Ghana Population and Housing Census show that 53.6% and 34.9% of households in rural and urban areas respectively do not have improved toilet facilities [35]. It is imperative to understand the differences between rural and urban settings, child’s characteristics and caregivers’ behaviour and characteristics that might influence unsafe stool disposal. This study examines factors that influence safe childhood stool disposal independently in either rural and urban areas of residence among children under two years in Ghana. The findings of this study would inform policy planners and decision-makers to adopt appropriate strategies in either rural and urban areas aimed at improving hygiene practices and reducing eventually child mortality. Safe disposal of young children’s stool is essential for children’s health, as it prevents oral-faecal diseases, reduces exposure to hazardous microorganisms and the burden of preventable diseases, which can have severe consequences for children’s health. The study findings will benefit households and communities by reducing the risk of waterborne diseases and promote healthier living, leading to a cleaner and more sustainable environment. For instance, identifying the barriers in rural areas could inform targeted interventions to improve sanitation practices, potentially reducing the spread of diseases.

## Methods

### Study design and description

This study used a nationally representative cross-sectional data from the sixth round of the Ghana Multiple Indicator Survey (MICS) conducted in 2017/18. The MICS used a multi-stage stratified sampling design. The first stage involved selecting primary sampling units or clusters stratified by region and rural-urban status. The selected clusters were listed to obtain the total number of households from which a systematic sample was obtained for the interview. This study is restricted to children under two years; hence, the children’s and women’s files were merged and yielded a sub-sample size of 3,476 [36]. Informed consent was obtained from all participants in the study. Participation in the survey was completely voluntary. Every respondent was made aware of the voluntary nature of the participation as well as the information’s anonymity and confidentiality. Respondents were also made aware of their right to discontinue the interview at any moment and to refuse to answer any or all of the questions [36].

### Measurement of variables

The dependent variable for this study is the safe disposal of children under two years faeces, categorised as “safe disposal of stools” and “unsafe disposal of stools”. During the survey, mothers or caregivers of these children were asked, “what was done to dispose of children’s stools?”. The responses included: “child used toilet/latrine”, “put/rinsed into toilet or latrine”, “put/rinsed into drain or ditch”, “buried”, “left in the open”, and “thrown into the garbage (solid waste)”, The outcome variable was grouped into safe and unsafe. Responses such as “child used toilet/latrine”, “put/rinsed into toilet or latrine” and “buried” were classified as “safe disposal of stools” in tandem with the WHO definition and classification of stool disposal [37]. On the other hand, “put/rinsed into drain or ditch”, left in the open”, and “thrown into the garbage (solid waste) were classified as unsafe disposal of stools. A binary variable was created, where “1” represents safe disposal and “0” represents unsafe disposal.

#### Independent variables

The explanatory variables included in the analysis were identified based on the literature reviewed. These are child characteristics [22, 30, 31, 38–40], caregiver characteristics [17, 41–45], and household factors [12, 14, 37, 46–48]. The child characteristics include the age of the child in months (0–5 months, 6–11 months, 12–17 months, and 18–23 months) and sex of the child (male and female).. Socio-demographic characteristics of the caregiver/mother were their ages in completed years, highest educational level (No education, primary, JSS/JHS/Middle and Higher), and marital status (in union, not in union), listen to radio (yes and no), watch television (yes and no), and place of residence (rural and urban). In addition, the household factors are household socio-economic status (rich, average and poor), ecological zone (coastal, middle and savanna), main source of drinking water classified as (improved and unimproved), and sanitation categories grouped into (improved and unimproved); based on the WHO categorisation of improved and unimproved water and sanitation sources [37, 46].

### Data analysis

The variables were examined using descriptive statistics to help situate the work in context, and Pearson chi-square test was performed to examine the relationship between each independent variable and the outcome variable. A binary logistic regression model was employed to explore the factors that are significantly associated with safe disposal of children’s stools. Three models were run using STATA version 16. The first model used the total data to examine the factors associated with the safe disposal of children’s stools accounting for the net effect of place of residence be it urban or rural and all other explanatory variables. The second and third models examined the urban-rural specific differentials.

## Results

### Background characteristics of study respondents

Table 1 shows that there is a slightly higher proportion of children less than two years being females (51%) and almost equal distribution across the various age groups in months in the data used in this study. The results further show that 61% of these children are found in households’ resident in rural areas. A higher proportion of caregivers were within 25–34 age group, followed by the 15–19 year group (33.9%). Nearly 46% of caregivers had lower than secondary education, while 8 out of 10 were in union. Over one-third (37.0%) belonged to the average household socio-economic status and resident in coastal or southern Ghana. In addition, 60% of caregivers listened to radio, and equal proportions (64.0%) watched television and had access to improved drinking water respectively. Furthermore, about 51% of caregivers had access to an improved toilet facility.

**Table 1:** Background Characteristics of Children and Caregivers

Indicators	Percentage (%)	Number
**Disposal of toilet**		
Safe	22.2	2705
Not safe	77.8	771
**Place of residence**		
Rural	61.1	2,125
Urban	38.9	1,351
**Sex**		
Male	49.4	1,719
Female	50.6	1,757
**Age of the child**		
0–5 months	25.6	891
6–11 months	26.0	904
12–17 months	23.7	823
18–23 months	24.7	858
**Age**		
15–24	33.9	1,177
25–34	43.5	1,511
35–49	22.7	788
**Education**		
No education	27.2	946
Primary	18.8	654
JSS/JHS/Middle	35.7	1,242
Secondary+	18.3	634
**Marital status**		
In union	82.9	2,883
Not in union	17.1	593
**Socio-economic status**		
Non-poor	32.5	1,131
Average	37.4	1,300
Poor	30.1	1,045
**Ecological zone**		
Coastal	37.1	1,288
Forest	31.7	1,102
Savanna	31.2	1,086
**Listening to Radio**		
No	39.9	1.388
Yes	60.1	2,088
**Watching Television**		
No	36.2	1,257
Yes	63.8	2,219
**Drinking water**		
Improved	73.8	2218
Unimproved	36.2	1,258
**Toilet**		
Improved	50.8	1,767
Unimproved	49.2	1,709
**Total**	**100.0**	**3476**

Table 2 shows test results of the associations between safe stool disposal and each of the independent variables at *p* < 0.05%. The results show that barely 1 in 4 (22.2%) children less than two years stools were safely disposed of. The age of the child is the only explanatory variable that had a statistically significant association with safe stool disposal in all three data sets (total, urban and rural). Safe stool disposal had a statistically significant association with the sex of the child, education, marital status, and socio-economic status of the caregivers; however, this significant association disappeared in the urban-rural divide. Unlike the child’s age, caregiver’s age had no significant association with safe stool disposal in all the three data sets.

**Table 2:** Test of Association between Background Characteristics and Safe Stool Disposal

	Total		Urban		Rural	
Indicators	Safe (%)	P value	Safe (%)	P value	Safe (%)	P value
**Place of residence**		*P* < 0.001				
Rural	26.2					
Urban	15.9					
**Sex**		*P* = 0.012		*P* = 0.102		*P* = 0.108
Male	24.0		18.4		27.8	
Female	20.4		13.4		24.7	
**Age of the child**		*P* < 0.001		*P* < 0.001		*P* < 0.001
0–5 months	10.2		6.7		12.5	
6–11 months	21.1		12.7		26.2	
12–17 months	24.9		16.9		30.9	
18–23 months	33.1		28.4		35.9	
**Caregiver’s Age**		*P* = 0.811		*P* = 0.862		*P* = 0.419
15–24	21.8		15.1		25.0	
25–34	22.7		16.2		27.7	
35–49	21.7		16.4		25.4	
**Education**		*P* < 0.003		*P* = 0.139		*P* = 0.137
No education	23.8		21.3		24.5	
Primary	23.2		16.1		29.1	
JSS/JHS/Middle	21.9		14.5		27.3	
Secondary+	17.2		15.1		21.8	
**Marital status**		*P* = 0.044		*P* = 0.153		*P* = 0.180
In union	22.8		16.6		26.7	
Not in union	19.1		12.9		23.3	
**Socio-economic status**		*P* < 0.001		*P* = 0.018		*P* = 0.929
Non-poor	16.6		13.8		25.8	
Average	24.5		19.8		26.6	
Poor	25.4		19.3		25.9	
**Ecological zone**		*P* = 0.216		*P* = 0.502		*P* < 0.001
Coastal	23.0		14.8		30.5	
Forest	23.0		17.3		28.4	
Savanna	20.4		15.6		21.5	
**Listening to Radio**		*P* < 0.001		*P* = 0.103		*P* < 0.001
No	19.2		13.5		21.7	
Yes	24.1		17.1		29.9	
**Watching Television**		*P* < 0.001		*P* = 0.023		*P* = 0.331
No	26.1		21.1		27.1	
Yes	19.9		14.9		25.5	
**Drinking water**		*P* = 0.148		*P* = 0.008		*P* = 0.107
Improved	22.9		18.4		25.2	
Unimproved	20.8		13.1		28.5	
**Toilet**		*P* = 0.014		*P* = 0.541		*P* < 0.001
Improved	23.9		16.3		33.3	
Unimproved	20.4		14.9		22.0	

Table 3 illustrates the results of the logistic regression models examining factors associated with safe child’s stool disposal in three data sets (combined, urban and rural). Results from Model 1 (combined data) revealed that place of residence, sex and age of child, listening to radio, socio-economic status, and marital status were significantly associated with safe child’s stool disposal. The study sought to determine whether the variables significant in the combined data would be significant at the disaggregated level. Model 2 (urban only) revealed that the sex and age of child, listening to radio, marital status and socio-economic status were significantly associated with safe child stool disposal independent of all other factors, while in Model 3 (rural only), the age of child, listening to radio, caregivers’ age, watching television, marital status, ecological zone and toilet facility were significantly associated with safe child stool disposal.

For model 2 (Urban only), female children’s stools were 28% less likely [aOR = 0.72; 95% CI = 0.53–0.98] to be disposed of safely compared to their male counterparts. The odds of safe disposal of children’s stools increase with age. For example, children between 18 and 23 months were 5.8 times more likely [aOR = 5.80; 95% CI = 3.51–9.58] to have their stools disposed of than those aged 0–5 months. Caregivers who listened to the radio were 54% more likely [aOR = 1.54; 95% CI = 1.06–2.21] to dispose of children’s stools safely than those who did not listen to the radio. Mothers and caregivers who were not in union were 45% less likely [aOR = 0.55; 95% CI = 0.35–0.88] to dispose of children’s stools safely than those in union.

Model 3 (Rural only), also shows a positive association between a child’s age and safe stool disposal. Listening to radio and marital status has a similar pattern to that observed in Model 2. Caregivers who watched television were 24% less likely [aOR = 0.76; 95% CI = 0.60–0.97] to dispose of children’s stools safely than those who did not watch television. Caregivers aged 25–34 years were 32% more likely [aOR = 1.32; 95% CI = 1.01–1.73] to dispose of their children stools safely than those aged 15–24 years. Stools of children in Savanna ecological zone were less likely [aOR = 0.65; 95% CI = 0.48–0.86] to be disposed of safely compared to children in the Southern zone. Children whose mothers had access to unimproved toilet facilities were 46% were less likely [aOR = 0.56; 95% CI = 0.44–0.70] to dispose of stools safely compared to those with improved sources.

**Table 3:** Factors associated with safe stool disposal in Ghana

	Model 1: Total		Model 2: Urban		Model 3: Rural	
Variables	aOR [95% C.I]	P > z	aOR [95% C.I]	P > z	aOR [95% C.I]	P > z
**Sex**						
Male (RC)						
Female	0.80 [0.67–0.94]	0.008	0.72 [0.53–0.98]	0.039	0.84 [0.69–1.03]	0.097
**Child’s age**						
0–5 months (RC)						
6–11 months	2.39 [1.82–3.14]	< 0.001	2.10 [1.22–3.61]	0.007	2.51 [1.82–3.46]	< 0.001
12–17 months	3.09 [2.35–4.07]	< 0.001	2.86 [1.70–4.80]	< 0.001	3.27 [2.36–4.54]	< 0.001
18–23 months	4.67 [3.57–6.09]	< 0.001	5.80 [3.51–9.58]	< 0.001	4.15 [3.02–5.69]	< 0.001
**Listening to Radio**						
No (RC)						
Yes	1.46 [1.21–1.76]	< 0.001	1.54 [1.06–2.21]	0.022	1.44 [1.16–1.79]	0.001
**Watching Television**						
No (RC)						
Yes	0.76 [0.62–0.94]	0.011	0.72 [0.47–1.10]	0.125	0.76 [0.60–0.97]	0.025
**Maternal age**						
15–24 (RC)						
25–34	1.23 [0.99–1.53]	0.066	1.07 [0.73–1.57]	0.736	1.32 [1.01–1.73]	0.042
35–49	1.22 [0.95–1.56]	0.115	1.34 [0.84–2.13]	0.215	1.17 [0.87–1.58]	0.285
**Education**						
Secondary + (RC)						
JHS/Middle	1.03 [0.79–1.35]	0.813	0.84 [0.58–1.24]	0.389	1.21 [0.81–1.80]	0.339
Primary	1.19 [0.88–1.61]	0.269	0.87 [0.52–1.44]	0.587	1.37 [0.90–2.09]	0.140
No education	1.25 [0.91–1.70]	0.164	1.29 [0.79–2.12]	0.310	1.32 [0.86–2.02]	0.198
**Marital status**						
In union (RC)						
Not in union	0.66 [0.52–0.86]	0.002	0.55 [0.35–0.88]	0.013	0.72 [0.53–0.97]	0.032
**Socio-economic status**						
Rich (RC)						
Average	1.49 [1.16–1.91]	0.002	1.51 [1.03–2.23]	0.036	1.31 [0.93–1.85]	0.123
Poor	1.49 [1.08–2.06]	0.014	1.64 [0.83–3.24]	0.152	1.36 [0.91–2.02]	0.132
**Ecological zone**						
Coastal (RC)						
Forest	0.93 [0.76–1.14]	0.508	1.09 [0.78–1.53]	0.615	0.84 [0.65–1.09]	0.195
Savanna	0.67 [0.53–0.86]	0.002	0.82 [0.50–1.35]	0.440	0.65 [0.48–0.86]	0.003
**Drinking water**						
Improved (RC)						
Unimproved	0.95 [0.79–1.14]	0.582	0.72 [0.51–1.01]	0.059	1.14 [0.91–1.42]	0.268
**Toilet**						
Improved (RC)						
Unimproved	0.61 [0.50–0.74]	< 0.001	0.74 [0.51–1.09	0.124	0.56 [0.44–0.70]	< 0.001
**Residence**						
Urban						
Rural	1.85 [1.48–2.30]	< 0.001				

## Discussion

This study used a nationally representative data to examine the factors associated with safe stool disposal in Ghana. The study found that only 22.2% of children under two years had stool safely disposed of. This is higher than what was recorded in India (21%) [19], but lower than Ethiopia (36.9%) [30], Papua New Guinea (47%) [40], Eswatini (58.2%) [44] and Nigeria (59.4%) [45]. The plausible reasons for these differences might be the age cut-offs and study time differences. There are several strategies, such as operation Clean Your Frontage, monthly nationwide clean-up exercise, the Ghana WASH Sector Development Programme (environmental sanitation and WASH in schools) and Community-Led Total Sanitation interventions in selected districts in Ghana, these strategies, according to Tchouchu and Ahenkan, [48], though have yielded much results yet not enough to meet SDG 6.2 and 3.9.2 by 2030.

The independent rural and urban analyses show that the proportion of safe disposal of children’s stool was higher in the rural analysis (26.2%) than the urban (15.9%). Our findings indicate that safe stool disposal was positively associated with a child’s age in rural and urban analysis. These results corroborate previous studies that have reported a positive association between a child’s age and the safe disposal of stools in Ethiopia [30], Bangladesh [22], and Malawi [31]. The probable reason could be that children’s less than six months of stools could be considered less harmful as they are smaller, smell-less and contain fewer visible food residues than children over six months [22 38]. Children aged 18 to 23 months usually can walk, talk and most likely be potty-trained. In addition, the common use of diapers, especially in urban areas, could contribute to why stools are not disposed of safely. Sahiledengle et al., [39] reported that there is a myth that young children’s stools are not particularly harmful in Low Middle-Income Countries, and that could also explain the unsafe disposal of stools at that age. In Ghana, due to the low access to improved toilet facilities [35], societies do not usually frown on open defecation among children. Hence, mothers/caregivers will do their best to dispose of the stool of children above 12 months more safely than those below 12 months.

Consistent with other studies [17, 41], listening to radio enables mothers/caregivers to get important health information about child waste disposal and its impact on children’s health. In addition, it has a great impact on behavioural change. For instance, Curtis et al., [41] reported that behavioural change programmes on hygiene promotion aired on the radio significantly impacted the safe disposal of stools. Irrespective of caregivers’ residents, these behavioural programmes tend to influence their attitude toward hygiene practices such as safe stool disposal.

The caregiver’s age in rural areas was associated with safe disposal of children’s stools. Mothers who were 25–34 years old were more likely to dispose of children’s stool safely than those who were 15–24 years. The findings of this study are consistent with other studies in Gambia [17] and Ethiopia [39]. The probable reason could be that there is mostly social support for childcare for young women in rural areas in Ghana [42]. Hence, as they age, these women may have learnt hygienic practices that could enable them to dispose of children’s stools safely, unlike their counterparts in urban areas.

Children whose mothers had access to unimproved toilet facilities were less likely to dispose of stools safely than those with improved toilet facilities for the total data and rural analysis. The findings of this study are consistent with other studies that have reported the safe disposal of children’s stool among those with improved sanitation [42–43]. Evidence shows that improving sanitation requires an individual to practice safe disposal of children’s stools, though it is insufficient. Caregivers with improved sanitation will adopt measures to practice good hygiene and make their environment clean [43]. In addition, Sara and Graham, [47] explained that ownership of improved sanitation motivates people to adopt safe hygienic practices. Therefore, there is a need to encourage households to have improved sanitation to facilitate safe disposal of stools. In Ghana, evidence shows that rural areas dominate unimproved toilet facilities [35].

This study’s factors associated with safe disposal support the socio-ecological framework. However, the separate rural and urban analysis reveals different factors supporting the framework at different levels. Analyses of the urban data show that child characteristics (sex and age) is the most significant variables, while in the rural analyses, the age of child, listening to the radio and toilet facility were most significant.

### Strength and limitation

This study used cross-sectional data and acknowledges that it makes it difficult to establish causal inferences between the independent and dependent variables. The study was delimited to only variables available in the dataset. Other important variables that potentially impact the safe disposal of stools, such as knowledge about child stool disposal, mothers’ hygiene practices, community factors, child care support, and others, were not part of the study variables. Notwithstanding, the study’s findings are very relevant towards implementing policies that will ensure the safe disposal of stools.

## Conclusion

Although safe disposal of children’s stool is low in Ghana, it is higher in rural than urban Ghana. This result revealed that different factors were significant in the rural and urban analysis, though few similar factors were significant in both rural and urban analysis. The split analysis shows that different policies are required to address child stool disposal among urban residents differently than rural residents. In urban areas, there is a need to have more targeted behavioural change programmes on sanitations on radio. Women not in a union should be targeted for intervention or assistance in supporting them to dispose of children stool safely. More sanitation programmes on radio and television should be aired in rural areas, and special attention should be given to the northern zone, unmarried women, young caregivers, and those without improved sanitation for an intervention to guide them in disposing of stools safely.

## Data Availability

The datasets generated and/or analysed during the current study are available in the Ghana Statistical Service database at the repository; https://www.statsghana.gov.gh/gssdatadownloadspage.php.
